# Edison Current in Cataphoresis

**Published:** 1897-03

**Authors:** L. E. Custer

**Affiliations:** Dayton, O.


ARTICLE IV.

### EDISON CURRENT IN CATAPHORESIS.

BY L. E. CUSTER, B. S., D. D. S., DAYTON, O.

In a former paper on cataphoresis read before the Mississippi Valley Dental Society, it was stated that the 110 volt Edison current was as good for cataphoric purposes as a battery current, and it was recommended on account of the convenience That statement was made from the practical experience in the use of the current as supplied in Dayton, O. Since that time the writer has had occasion to operate with a variety of commercial currents throughout the country. In Marshalltown, Iowa, the 500 volt current was used, but in all the other instances the 110 volt Edison. The experience with some of the currents was so different from that at Dayton that I was at a loss for a satisfactory explanation. In some there was so much pain in the application of the current that the operation could not be called a success so far as being painless was concerned. When the appliances were perfectly quiet the patient would from time to time experience sudden and severe shocks of pain, much as if the current had been broken.

It was also shown in the paper that to produce cataphoresis painlessly the current pressure, when supplied by either a dynamo or battery, must be steadily maintained. It will not do to suddenly raise or lower the voltage on the main of the street current. Neither is it allowable to cut in or out a cell or a number of cells of a battery. By my experience with the various currents I was led to suspect that in those instances where there was this periodic pain to the patient there must be a sudden and wide variation in the voltage, although this did not show itself upon the voltmeter by more than a slight movement of the index finger. Subsequent observation and experimentation has practically substantiated the above belief, and I feel safe in saying that in those instances where operators are using the 110 volt current with intermittent pain to the patient, the anode being held quiet, that it is due to a fluctation in the pressure of the main current. The fluctuation is usually a lowering of the voltage and so quickly done that it is not noticeable on the ordinary voltmeters in use. It is not a drop of a few volts but of quite a number, possibly of twenty or thirty at times.

The Edison constant current, the most practical form of commercial current to use for cataphcresis, is furnished us at a pressure of about 110 volts. This current as is well known, is produced by the rotation of an armature between the poles of an electro-magnet. In order to maintain 110 volts the armature must rotate at a given speed, the armature must have a certain number of windings and there must be a certain magnetic flux. If any of the above three conditions should vary there would be a variation in the voltage of the outgoing current. It is by means of the last named that the voltage is most commonly controlled, or rather maintained, because it can be done by means of the rheostat. It is not practical to vary the speed of the engine, nor is it practical to change the windings of the dynamo, but the magnetism of the field can be easily varied by an instrument in common use, the rheostat. In a large plant where the manufacture and consumption of current is large, as for instance a city plant, where a number of dynamos are feeding into common mains and where the output is always large, the cutting in or out of current for a building, or perhaps for a whole square, is scarcely noticeable, and the average voltage is easily and carefully maintained, whereas in small plants which supply but a building, or but a few at most, often with imperfect regulators and inefficient attendance, the variation in voltage is much more marked. The voltage of a city plant may vary 5 or 8 volts during the day, but this variation has been going on very gradually. But in the smaller plants the variation comes on instantly.

When a dynamo has once been placed in operation, its armature is expected to revolve at a certain speed at all times in order to produce and maintain a steady voltage. It is necessary to increase the belt pull on the armature as the output of current is increased. It is not simply a matter of revolving the weight of the armature on its axis against the air friction and the friction of its bearings, but as the load or output increases magnetism also increases and the armature becomes a dead weight, like revolving a fan in a vessel of water, and as the output still further increases the water theoretically begins to thicken. Now this is a dead weight and if the belt pull were to suddenly cease, the armature, whose momentum is neutralized by the magnetism, would stop. It would not entirely stop, it would at least lessen its speed very considerably. This all occurs so quickly that the eye cannot perceive it and the ordinary voltmeter index has not had time to record it. All that is noticeable to the eye is a slight movement of the index finger. Now if this current were used for cataphoresis at the time of this sudden drop of the voltage, which is not measured on the meter, it is very distinctly felt by the patient, because it would be practically the same thing as lifting the anode from the tooth. In a large plant where a number of dynamos are feeding into the same lines the slipping of a belt on one is scarcely noticeable, but in a small plant this is certainly the case.

A frequent cause of the variation of voltage in small plants is that the engines and dynamos are often too small for the work put upon them. No engine can run smoothly when it is always put to the highest limit. It is the one which has reserve that can be regulated to meet the varying demands. A still less satisfactory source, so far as steady pressure is concerned, is the gas engine. While this is especially adapted for small plants, the smaller the engine the more unstead the electrical output.

Another cause of variation of voltage is found where the dentist does not get his current direct from the main line or is quite near some person who is using current from the same wires periodically. In many large buildings the wires enter the basement and ramify throughout the entire structure, giving off branches to each floor and these in turn to the different suites. Perhaps the elevators are operated from the same wires. It is a commonly observed fact that if a lamp is steadily burning and another near by be turned on, if the conductors are small, the first lamp is seen to slightly dim. The same thing occurs when the operator is producing cataphoresis and a neighbor turns on or shuts off current for his engine, lamp or oven.

The current used for lighting is always consumed at a steady rate and mostly at night, but that for motors is periodic, and it is unfortunate that this is during the daytime and when the dentist operates his cataphoric appliance. For this reason it is also important that the dentist secure his current from main lines if possible.

Occasionally the Edison wires are crossed by the trolley. If there are no grounds on the dentist's line, nothing further than a small raise in the voltage will be noticeable and but a slight shock is felt by the patient, but should his wires be in contact with a water or gas pipe the results would be more serious. On a well-kept system the heavy voltage is immediately dissipated in the many outlets and nothing serious will result.

The practical conclusions to be drawn are these. The 110 volt Edison current may be used satisfactorily and with as good results as a battery current where it is furnished by a well equipped and not overloaded plant. The dentist should connect direct to the main line whenever possible and not use current for other purposes while producing cataphoresis. He should not be on the same circuit with large motors which operate intermittently and should see that his wiring is perfectly insulated and not in danger of a ground.—*Ohio Dental Journal.*

